# Association of sensitization to food and inhalant allergens in patients of asthma and rhinitis

**DOI:** 10.1186/2045-7022-1-S1-P119

**Published:** 2011-08-12

**Authors:** Raj Kumar

**Affiliations:** 1 University of Delhi, Vallabhbhai Patel Chest Institute, Department of Respiratory Allergy & Applied Immunology, and Department of Pulmonary Medicine, Dehli, India

## Background and objective

Recent estimates suggest that IgE- mediated food allergy affects 6 %-8 % children and 3%-4 % adults . The present study was conducted to investigate the association of sensitization to food and inhalant allergens in patients of asthma and rhinitis.

## Methods

Diagnosed patients of Asthma and rhinitis were evaluated for sensitization to food and inhalant allergens. Patients (12-62 years) were screened using standard questionnaire. The skin prick-tested (SPT) was done with common foods and aeroallergens in history positive patients of food allergy.

## Results

Of 1860 patients screened, 1097 (58.9 %) gave history of food allergy. Of the history positive patients 470 were skin prick tested . 29.3 % (138/470) exhibited positive reactions to one or more food(s). Rice elicited SPT positive reaction in maximum ( 6.2 %) cases followed by blackgram (5.9 %), lentil (5.5 %), citrus fruits (5.3 %), pea (3.8 %), maize (3.8%) and banana (3.6 %). Among food sensitised cases 35.5% ( 49/138 ) of patients also showed positive skin reaction to one or more pollen extracts. A majority of the food sensitized patients (52.1 %) were skin test positive to insect allergens while only 14.7 % showed positivity with fungal extracts. Positive skin reaction with food co-existed with positive SPT to insects extracts. Sensitisation to food allergen (potential food allergy) was significantly associated with asthma(P=0.0065) whereas inhalant allergens (pollen, fungal and insects) were strongly related to rhinitis (P<0.01). However sensitisation with pollens was less common in patients of asthma with rhinitis. Sensitisation to food allergens was significantly associated with asthma alone ( P=0.0001). But food sensitivity is significantly less common in cases of asthma associated with rhinitis (P<0.028). Correlations between sensitisation to aeroallergens and ten common foods were analyzed.

## Conclusions

The results suggest that concommittant sensitisations to food with pollen and insect may enhance the risk of asthma and rhinitis or contribute towards exacerbation of symptoms. The synergistic action of these factors may influence the development and progression of atopic manifestation.

